# The function of RAD52 N-terminal domain is essential for viability of BRCA-deficient cells

**DOI:** 10.1093/nar/gkaa1145

**Published:** 2020-12-04

**Authors:** Kritika Hanamshet, Alexander V Mazin

**Affiliations:** Department of Biochemistry and Molecular Biology, Drexel University College of Medicine, Philadelphia, PA 19102, USA; Department of Biochemistry and Molecular Biology, Drexel University College of Medicine, Philadelphia, PA 19102, USA

## Abstract

RAD52 is a member of the homologous recombination pathway that is important for survival of BRCA-deficient cells. Inhibition of RAD52 leads to lethality in BRCA-deficient cells. However, the exact mechanism of how RAD52 contributes to viability of BRCA-deficient cells remains unknown. Two major activities of RAD52 were previously identified: DNA or RNA pairing, which includes DNA/RNA annealing and strand exchange, and mediator, which is to assist RAD51 loading on RPA-covered ssDNA. Here, we report that the N-terminal domain (NTD) of RAD52 devoid of the potential mediator function is essential for maintaining viability of BRCA-deficient cells owing to its ability to promote DNA/RNA pairing. We show that RAD52 NTD forms nuclear foci upon DNA damage in BRCA-deficient human cells and promotes DNA double-strand break repair through two pathways: homology-directed repair (HDR) and single-strand annealing (SSA). Furthermore, we show that mutations in the RAD52 NTD that disrupt these activities fail to maintain viability of BRCA-deficient cells.

## INTRODUCTION

Homologous Recombination (HR) is a process crucial for the repair of DNA double-strand breaks (DSBs) and maintenance of genomic integrity. HR promotes DSB repair in an accurate manner using homologous DNA sequences as a template (1,2). Proteins responsible for HR were initially identified in *Saccharomyces cerevisiae* during genetic screening for mutants sensitive to IR and collectively defined as *RAD52* epistasis group (3). Among the members of this group, *rad52* showed the strongest phenotype indicating an important role of Rad52 protein in all HR events.

Biochemical studies have identified several functions of Rad52; it can promote ssDNA annealing between complimentary strands, the activity essential for the single-strand annealing (SSA) pathway of DSB repair (4–7). Rad52 can also promote DNA strand exchange (DNA pairing) between ssDNA and supercoiled plasmid dsDNA resulting in formation of D-loop mimicking the *in vivo* strand invasion process (8–10), sharing this activity with Rad51, the major human recombinase. Rad52 also plays a role in RNA mediated DNA repair through its two activities: ssRNA-ssDNA annealing (11) and inverse RNA strand exchange (IRSE) (12). In IRSE, Rad52 forms a complex with dsDNA and promotes strand exchange with homologous RNA. Apart from the DNA/RNA pairing and annealing activities, in yeast Rad52 functions as a Rad51 mediator wherein it loads Rad51 on RPA-covered ssDNA (13–17). Genetic studies in yeast demonstrated that that the mediator function of Rad52 plays a dominant role in DNA damage repair.

Unlike yeast *rad52* null mutants, *Rad52* knock-out mice show no or mild deficiency in DNA repair; they are viable, fertile, and do not display cancer predisposition (18,19). Human RAD52 shares the biochemical activities with yeast Rad52 except that the mediator activity has not been yet demonstrated. It is thought that in mammalian cells, BRCA2 supplanted the mediator function of RAD52 (20,21). The role of mammalian RAD52 was hence thought to be of a lesser significance until Powell's group demonstrated that RAD52 is essential for viability of cells carrying mutations in *BRCA1*, *BRCA2* and several other HR genes (*PALB2* and five *RAD51* paralogs) (22,23). However, the mechanisms of this dependency of BRCA-deficient cells on RAD52 is not currently understood.

**Figure 1.**
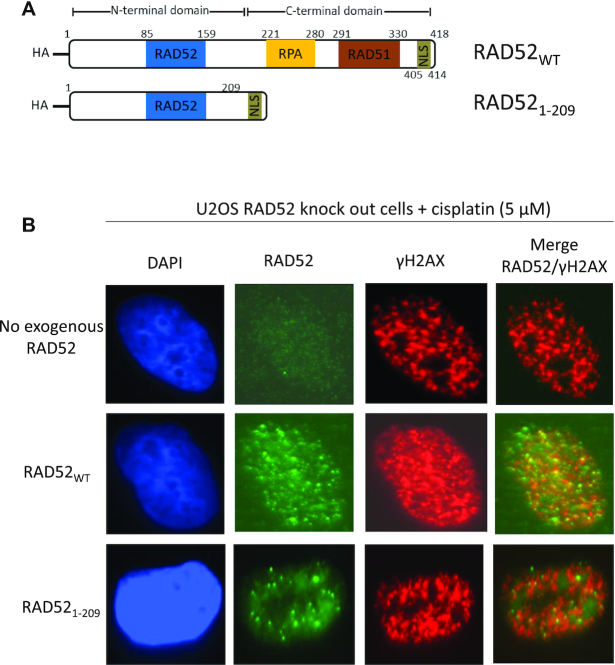
RAD52_1–209_ forms nuclear foci in response cisplatin treatment in U2OS cells. (**A**) The scheme of RAD52_WT_ and RAD52_1–209_ constructs containing HA-tag at the N-terminus. RAD52 NTD domain contains DNA binding region and a region for oligomerization (blue; 85–159 residues). RAD52 CTD contains RPA binding region (yellow; 221–280 residues), RAD51 binding region (brown; 291–330 residues) and nuclear localization signal (NLS) (green; 405–414 residues). 405–418 residues containing NLS was added to RAD52_1–209_ at the C-terminus. (**B**) Representative images of U2OS RAD52 knock-out (KO) cells stably expressing RAD52_WT_ or RAD52_1–209_ or no exogenous RAD52 stained with DAPI (blue), fluorescent anti-bodies for RAD52 (green) and γ-H2AX (red) 6 h after cisplatin (5 μM) treatment.

Structurally, human RAD52 consists of two equally sized domains: N-terminal domain (NTD), amino acid (aa) residues 1–209, and C-terminal domain (CTD), aa residues 210–418 (Figure 1A). These two domains show a remarkable separation of RAD52 functions. The NTD is responsible for RAD52 oligomerization, binding to ssDNA and dsDNA, and DNA/RNA pairing and annealing (12,18,24,25). The CTD is involved in interaction with RPA and RAD51 and contains a nuclear localization signal (26).

In this study, we investigated the role of the NTD (aa residues 1–209) of RAD52 and its encoded activities in maintenance of viability of BRCA-deficient cells. We found that RAD52_1–209_ alone is able to maintain the viability of both *BRCA1^−^^/^^−^* and *BRCA2^−^^/−^* cells. We also show that RAD52_1–209_ can promote homology-directed DSB repair (HDR) in BRCA-deficient cells, SSA, and form nuclear foci upon DNA damage. In contrast, RAD52 mutants that are deficient in DNA binding and DNA pairing activities but contain an intact CTD fail to support viability of BRCA-deficient cells and other functions of RAD52 in human cells. Taken together, our biochemical and cellular results indicate the critical role of the RAD52 NTD and the activities it encodes for viability of BRCA-deficient cells.

## MATERIALS AND METHODS

### Proteins and oligonucleotides

His-tagged human RAD52 and the RAD52 mutant proteins (NTD_1–209_, RAD52_YI65–66AA_ and RAD52_K102A/K133A_) were purified from *Escherichia coli* BL21 (DE3) cells (New England Biolabs). Briefly, cells were grown at 37°C to an O.D._600_ of 0.6–0.8, protein expression was induced by 1 mM isopropyl-1-thio-β-d-galactopyranoside for 4 h at 37°C. Cells were harvested by centrifugation (7000 × g) and stored at −80°C. All purification steps were carried out at 4°C. Cells (5–8 g) were thawed and resuspended in ten volumes of lysis buffer (50 mM KH_2_PO_4_ pH 7.5, 500 mM KCl, 10% sucrose, 10 mM 2-mercaptoethanol) supplemented with EDTA-free protease inhibitor cocktail (Roche Applied Science) and lysed by passing cell suspension twice through an EmulsiFlex-C5 (Avestin) at 15 000–20 000 psi. The crude extract was clarified by centrifugation (135 000 × g for 60 min) and passed through 0.45 μm filter. The filtrate was loaded onto a 5 ml HiTrap Ni^2+^ column (GE Healthcare). The column was washed extensively with buffer A (50 mM KH_2_PO_4_ pH 7.5, 500 mM KCl, 10% glycerol, 10 mM 2-mercaptoethanol) supplemented with 50 mM imidazole. RAD52 or RAD52 mutant proteins were eluted with buffer A supplemented with 500 mM imidazole. The eluate was then fractionated in a Superdex 200 column (58 ml) equilibrated with buffer A. The fractions containing RAD52 or RAD52 mutant proteins were pooled, diluted five times with buffer B (50 mM KH_2_PO_4_ pH 7.5, 10% glycerol, 10 mM 2-mercaptoethanol) and loaded on to a 1 ml Heparin column (GE Healthcare), equilibrated with buffer B supplemented with 100 mM KCl. The proteins were eluted with a 20 ml gradient of KCl (100–1000 mM) in buffer B. The fractions containing RAD52 or RAD52 mutant proteins were analyzed for nuclease contamination, pooled, and stored in small aliquots at −80 °C. Human RPA was purified as described by Henricksen et al., 1994, JBC, 269: 11121-32.

The oligonucleotides (Supplementary Table S1) were purchased from IDT Inc. and further purified by electrophoresis in polyacrylamide gels containing 50% urea (27). dsDNA substrates were prepared by annealing of equimolar (molecules) amounts of complementary oligonucleotides. Oligonucleotides were labeled using [γ-^32^P] ATP and T4 polynucleotide kinase (New England Biolabs).

### D-loop assay

The nucleoprotein complexes were assembled by incubating RAD52_WT_ (450 nM), RAD52_1–209_ (800 nM), RAD52_YI65–66AA_ (450 nM), or RAD52_K102A/K133A_ (450 nM) with ^32^P-labeled ssDNA (no.160, 80-mer) (3 μM, nucleotides) in buffer A containing 25 mM Tris-acetate pH 7.5, 100 μg/ml BSA, 0.3 mM magnesium acetate, and 2 mM DTT for 15 min at 37°C. The optimal concentrations of RAD52 and RAD52 mutant proteins were determined in preliminary experiments. D-loop formation was initiated by addition of supercoiled pUC19 dsDNA (50 μM, nucleotides) at 37°C. Aliquots (10 μl) were withdrawn at indicated time points and deproteinized by incubation with stop buffer containing 1.5% SDS, 0.8 mg/ml proteinase K, 7% glycerol and 0.01% bromophenol blue for 15 min at 37°C. Samples were analyzed by electrophoresis in 1% agarose gels in TAE buffer (40 mM Tris acetate pH 8.3, and 1 mM EDTA) at 5 V/cm for 1.5 h at room temperature. The gels were dried on Amersham Hybond-N+ membrane (GE Healthcare) and analyzed using a Typhoon FLA 7000 biomolecular imager (GE Healthcare). For experiments with RAD52 inhibitor D-I03, RAD52 was first incubated with indicated concentrations of D-I03 in buffer A for 15 min at 37°C. DMSO (2%) was used as a control. Next, ^32^P-labeled ssDNA (no.160, 80-mer) (3 μM, nucleotides) was added and incubated for 15 min at 37°C. Supercoiled pUC19 dsDNA (50 μM, nucleotides) was next added and incubated for 30 min at 37°C. The reactions were stopped and deproteinized by addition of stop buffer 15 min at 37°C. Samples were analyzed as mentioned above.

### Annealing assay

The fluorescein-labeled ssDNA (no. 337-F, 60-mer) (0.5 nM, molecules) was pre-incubated with human RPA (1 nM) or RPA dilution buffer for 5 min in buffer containing 25 mM Tris-acetate pH 7.5, 100 μg/ml BSA, 5 mM Magnesium acetate, 100 mM NaCl and 2 mM DTT at 30°C. Then RAD52 nucleoprotein complexes were assembled by adding RAD52_WT_ (10 nM), RAD52_1–209_ (40 nM), RAD52_YI65–66AA_ (10 nM) or RAD52_K102A/K133A_ (10 nM) to the reaction and incubating for 5 min at 30°C. The optimal concentrations of RAD52 and RAD52 mutant proteins were determined in preliminary experiments. Annealing was initiated by addition of complementary ssDNA carrying a Black Hole Quencher 1 (BHQ) group (no.1337-BHQ, 39-mer) (0.5 nM, molecules) and the fluorescence intensity was measured in a 4-mm quartz cuvette (Starna Cells) using a FluoroMax-3 (Horiba) fluorimeter with 492 nm excitation and 520 nm emission wavelength at 30°C. Annealing was measured by the amount of quenching.

### Inverse RNA strand exchange assay

The nucleoprotein complexes were assembled by incubating RAD52_WT_ (900 nM), RAD52_1–209_ (1.4 μM), RAD52_YI65–66AA_ (900 nM), or RAD52_K102A/K133A_ (900 nM) with a 30 nucleotide-tailed dsDNA (nos. 1/117; 68.6 nM molecules) in buffer containing 25 mM Tris-acetate pH 7.5, 100 μg/ml BSA, 2 mM magnesium acetate, and 2 mM DTT at 37°C for 15 min. The optimal concentrations of RAD52 and RAD52 mutant proteins were determined in preliminary experiments. The reactions were initiated by addition of RNA (no. 2R; 205.8 nM, molecules). Aliquots (10 μl) were withdrawn at indicated time points and deproteinized by incubation with 1% SDS, 1.6 mg/ml proteinase K, 7% glycerol and 0.01% bromophenol blue for 15 min at 37°C. Samples were analyzed by electrophoresis in 10% polyacrylamide gels (acrylamide/*N*,*N*'-methylenebisacrylamide 17:1) in TBE buffer (89 mM Tris, 89 mM boric acid and 1 mM EDTA, pH 8.3) at 13 V/cm for 1.5 h at room temperature. The gels were dried on Amersham Hybond-N+ membrane (GE Healthcare) and analyzed using Typhoon FLA 7000 biomolecular imager (GE Healthcare).

### Cell culture

BRCA1-deficient MDA-MB-436 and BRCA1-proficient counterpart MDA-MB-436 cell lines were obtained from Dr Neil Joshnson (28). BRCA1-deficient HCC1937 cells were obtained from ATCC (Catalog no. CRL-2336). Both MDA-MB-436 and HCC1937 were cultured in RPMI-1640 (ATCC, Catalog no. 30-2001) supplemented with 10% FBS (Gibco, Catalog no. 16000044), penicillin (100 units/ml) and streptomycin (100 μg/ml) (Sigma). BRCA2-deficient CAPAN-1 cells were obtained from ATCC (Catalog no. HTB-79) and were cultured in IMDM (ATCC, Catalog no. 30-2005) supplemented with 20% FBS, penicillin (100 units/ml) and streptomycin (100 μg/ml). U2OS SSA-GFP cells were obtained from Dr Jeremy Stark (29). HEK293T cells were obtained from ATCC (Catalog no. CRL-3216). Both U2OS-SSA-GFP and HEK293T were cultured in DMEM (Sigma, Catalog no. D6429) supplemented with 10% FBS, penicillin (100 units/ml) and streptomycin (100 μg/ml). All cell lines were maintained at 37°C in humidified atmosphere containing 5% CO_2_ and tested for mycoplasma contamination routinely using PCR Mycoplasma Detection Kit (Applied Biological Materials Inc).

### RAD52 knock-out in U2OS cells

The PX330 vectors with single-guide RNAs (sgRNAs no. L1 and R1, Supplementary Table S1) targeting *RAD52* were a kind gift from Dr Li Lan (30). The PX330 vectors (1.5 μg each) were transfected in U2OS cells (plated previous day at 3 × 10^5^ cells/well in a 6-well plate; 2 ml antibiotic-free medium) with TransIT-LT1 (9 μl) (Mirus). After 24 h, cells were trypsinized, and single cells were seeded in 96-well plates to obtain single colonies. After 10 days, single colonies were transferred to 24-well plates and grown for about 1 week before genome DNA extraction (Zymo Research, D3006). Screening for *RAD52* deletion was done by PCR (*RAD52* check forward and reverse primer, Supplementary Table S1), and then knock-out of RAD52 protein was verified by western blots.

### Generation of stable cell lines expressing RAD52

HA-tagged RAD52_WT_ or RAD52 mutant constructs were generated using plenti-CMV-neo vector (Addgene, #17447) by replacing GFP gene with RAD52_WT_ or RAD52 mutants. For RAD52_1–209_ construct, residues 405–418 containing the nuclear localization signal (405–414) were added after 209th residue. For RAD52_YI65–66AA_ and RAD52_K102A/K133A_, Tyr65-Ile66 and Lys102/Lys133 were mutated to alanine residues, respectively. The plenti-CMV-neo vectors with RAD52 constructs (25 μg) were co-transfected with packaging plasmids (pCMV-dR8.74, 6.5 μg and pMD2-VSVG, 3.5 μg) into HEK293T cells (plated previous day at 6 × 10^6^ cells/10 cm plate; 10 ml medium) using TransIT-LT1 (70 μl). Culture medium was changed 6 h post-transfection with DMEM medium containing 10% FBS and 1% BSA. Medium containing viral particles was collected after 24 and 48 h and filtered through 0.45 μm filter and stored at –80 °C. For stable integration, cells were infected overnight with viral particles (200 μl of each 24 h and 48 h timepoint) in 1.6 ml culture medium containing 8 μg/ml polybrene. Medium was changed with regular DMEM–10% FBS media after 24 h. Following day, MDA-MB-436, HCC1937, CAPAN-1 and U2OS cells were selected with G418 antibiotics at concentrations 0.75, 0.5, 1 and 0.5 mg/ml, respectively, for 10–14 days. The medium was changed by fresh one containing G418 antibiotic every 3 days. Stable cell lines were maintained in medium containing G418.

### RAD52 knock-down with shRNA

pLKO vector expressing shRNA targeting 3′UTR of RAD52 (shRAD52) (Sigma, SHCLND-NM_134424.2–1462s21c1) or Non-Target shRNA control (shCtrl) (Sigma, SHC202) were used. The oligonucleotide sequences used for shRNA are shown in Supplementary Table S1. Viral particles for both shRAD52 and shCtrl were made as mentioned above using HEK293T cells. For stable integration of shRNA, cells were infected with viral particles in culture medium containing 8 μg/ml polybrene. Culture medium was changed after 24 h. Next day, cells were selected using 2 μg /ml (MDA-MB-436) or 1 μg/ml (HCC1937 and CAPAN1) of puromycin for 7 days, with medium change every 2 days.

### Colony formation assay

Cells expressing RAD52 WT and mutants were treated with shRAD52 or shControl as mentioned above. After selecting with puromycin, the cells were trypsinized, counted using a TC20 automated cell counter (Bio-Rad), serially diluted, and seeded at concentration of 400 (MDA-MB-436) or 1000 (CAPAN1 and HCC1937) cells per well in a six-well plate and incubated for 14 days. Following incubation, cells were fixed with 100% methanol for 10 min at 4°C and then stained with 0.5% crystal violet solution in 25% methanol for 15 min. Colonies were counted using ImageJ and normalized to cells expressing empty vector (EV) and treated with shControl. shRNA viral infections were done each time for each independent experiment.

### Single strand annealing in U2OS cells

U2OS-SSA-GFP (with RAD52 KO or expressing HA-RAD52) were plated at a density of 2 × 10^5^ cells/well in six-well plates. After 22 h, cells were washed with 1× PBS and further incubated for 2 h in antibiotic-free DMEM-10% FBS. Cells were transfected with pCBASce (0.8 μg) expressing I-SceI endonuclease or, pMX-GFP (0.4 μg) plasmids using Lipofectamine 2000 (3.6 μl). pMX-GFP plasmid was used as a transfection control. After 3 h of transfection, cells were washed with antibiotic-free DMEM–10% FBS. Then, cells were incubated in DMEM–10% FBS supplemented with antibiotics for 72 h. In each well, cells were washed with 1× PBS (phosphate buffered saline), trypsinized, and fixed with 3.3% formaldehyde. Fixed cells were kept on ice. The yield of GFP+ positive cells was measured by flow cytometry using Guava EasyCyte PRO (EMD Millipore) and normalized to transfection efficiency (pMX-GFP).

### Immunofluorescence staining

Cells were plated at a concentration of 3–5 × 10^5^ on glass coverslips in a six-well plate. Next day, the cells were treated with cisplatin (5 μM for U2OS cells and 10 μM for MDA-MB-436 cells) for 6 h (U2OS cells) or 16 h (MD-MB-436 cells). Following treatment, coverslips were washed with 1× PBS and then incubated in buffer containing 25 mM HEPES, pH 7.5, 50 mM NaCl, 1 mM EDTA, 3 mM MgCl_2_, 300 mM Glucose and 0.5% Triton X-100 for 15 min on ice. Cells were then fixed in 3% formaldehyde and 2% sucrose in PBS for 10 min at room temperature and then washed three times with PBS, permeabilized by 0.5% Triton X-100 in PBS for 8 min, and then washed again three times with PBS. The coverslips were then incubated with blocking buffer (5% BSA (Sigma, A-7030) in 0.05% PBST (1× PBS and 0.05% Tween 20)) for 1 h at room temperature. Cells were then incubated with primary antibodies diluted in blocking buffer overnight at 4°C. Next day, the cells were washed three times with 0.05% PBST and incubated with secondary antibodies diluted in blocking buffer for 1 h at room temperature. Finally, they were washed three times by 0.05% PBST and mounted on slides using ProLong™ Gold Antifade Mountant with DAPI. The primary antibodies used for immunoassays are anti-RAD52 (Abcam-124971, 1:1000), anti-HA (Biolegend-901533, 1:500) and anti-γH2AX ser139 (JBW301, EMD Millipore 05-636, 1:20 000). The secondary antibodies are Goat anti-Mouse IgG1 Alexa flour 568 (Invitrogen-A21124, 1:1000) and Goat anti-Rabbit IgG Alexa flour 488 (Invitrogen-A11034, 1:1000). The images were captured using EVOS FL auto imaging system (Life Technologies).

### Homology directed repair (HDR) luciferase assay

The HDR luciferase reporter vectors (gWiz.Lux-5′-3′ Luc) and gWiz Luciferase (gWiz.Lux) were a kind gift from Dr Ryan Jenson (31). MDA-MB-436 cells were seeded in a six-well plate at a concentration of 2 × 10^5^ cells/well for 20 h. Then, cells were transfected with 200 ng of gWiz.Lux-5′-3′Luc vector and 1 μg of the I-SceI expressing vector using Lipofectamine 2000 (3.6 μl). gWiz.Lux-5′-3′Luc or gWiz.Lux (200 ng) vector were transfected alone and used as negative and positive controls, respectively. After 72 h, cells were washed with 1× PBS and then incubated with 1× PBS for 10 min on ice. Cells were lysed with 200 μl of lysis buffer containing 50 mM HEPES (pH 7.5), 250 mM NaCl, 0.5 mM EDTA, 1 mM DTT and 1× complete EDTA-free protease inhibitor cocktail (Roche Applied Science) for 20 min on ice and then centrifuged for 15 min at 16 000 × g at 4°C. Luminescence was measured using an integration time of 1 s with 40 μl of the lysate plus 100 μl of luciferin substrate (One-Glo luciferase assay, Promega). The raw data was normalized to both cellular protein levels and gWiz.Lux for transfection control. For experiments with RAD52 inhibitor D-I03, after washing off the transfection complexes, media with D-I03 (50 μM) or DMSO (1%) was added for 72 h and then followed by steps described above.

### Western blotting

Cell pellets were washed with cold 1× PBS and then lysed in buffer containing 25 mM Tris–HCl, pH 7.5, 120 mM NaCl, 0.5% NP-40, 1 mM EDTA, 1 mM DTT and 1× complete EDTA-free protease inhibitor cocktail (Roche Applied Science) for 30 min on ice. Lysates were then centrifuged for 15 min at 16 000× g at 4°C, and supernatant was collected. Protein concentration was measured using Bradford's reagent (Bio-Rad), and 50–100 μg of protein sample was loaded on 15% SDS-polyacrylamide gels (acrylamide/*N*,*N*'-methylenebisacrylamide 30:0.8) after heating for 5 min at 95°C in 1× Laemmli buffer (50 mM Tris–HCl pH 6.8, 10% glycerol, 1% β-mercaptoethanol, 2% SDS and 0.01% bromophenol blue). After electrophoresis, samples were transferred to a PVDF membrane. The membrane was blocked with 5% non-fat milk in 1× TBST buffer (10 mM Tris–HCl (pH 8.0), 150 mM NaCl and 0.1% Tween20) for 1 h and then incubated with indicated primary antibody diluted in 5% bovine serum albumin (BSA) in 1× TBST overnight at 4°C. Next day, the membrane was washed three times with 1× TBST and then incubated with horseradish peroxidase (HRP)-conjugated secondary antibody (Jackson Immunoresearch Laboratories Inc.) at 1:10 000 dilution in TBST buffer with 5% nonfat milk for 1 h at room temperature. The membrane was then washed six times with 1× TBST and then incubated in west pico reagent (Pierce) as described by manufacturer. Images were acquired using G:BOS XX6 (Syngene). The primary antibodies used are RAD52 (Abcam 124971, 1:1000), actin (Sigma, 1:10000) and nucleolin (Santacruz-17826, 1:1000).

### Quantification and statistical analysis

For conducting statistical analysis, Prism 8.4.0 GraphPad software was used. Number of repeats (*n*), *P*-value and type of statistical test used for each graph is specified in each figure legend.

## RESULTS

### RAD52_1-209_ forms nuclear foci in human cells in response to DNA damage

In response to DNA damage, RAD52 forms nuclear foci that are thought to play a role in DNA repair. Here, we wanted to examine whether the NTD of RAD52 maintains this ability. We constructed the RAD52_1–209_ comprising of first 209 aa residues with HA-tag at its amino terminal end (Figure 1A). For proper localization of RAD52_1–209_ to the nucleus, 405–418 residues from the CTD containing the nuclear localization signal (405–414 aa) were incorporated after 209th aa residue of RAD52. Using CRISPR-cas9 we first knocked out endogenous RAD52 in U2OS cells and then with lentiviral system, stably integrated exogenous HA-tagged RAD52_1–209_, or HA-tagged RAD52_WT_ as a control (Supplementary Figure S1A and B). To induce DNA damage U2OS RAD52 KO cells expressing exogenous RAD52_1–209_ or RAD52_WT_ were treated with cisplatin (5 μM), a DNA damaging agent, for 6 h. The foci were visualized using anti-RAD52 antibody and as a control, γH2AX foci were used as a DNA damage marker. We noticed that both RAD52_WT_ and RAD52_1–209_ formed foci that co-localized with γH2AX foci (Figure 1B). We confirmed this result in another cell line, MDA-MB-436, where exogenous RAD52_1–209_ formed foci that co-localized with γH2AX foci (Supplementary Figure S1C). To ensure that the foci are from the exogenous RAD52_1–209_ protein we also probed the cells with HA-antibody. We saw complete co-localization of RAD52 and HA antibodies (Supplementary Figure S1D). Taken together, our results demonstrate that RAD52_1–209_, similar to the full-length RAD52, is proficient in formation of nuclear foci in human cells in response to DNA damage.

### RAD52_1–209_ maintains viability of BRCA1- and BRCA2-deficient cells

**Figure 2.**
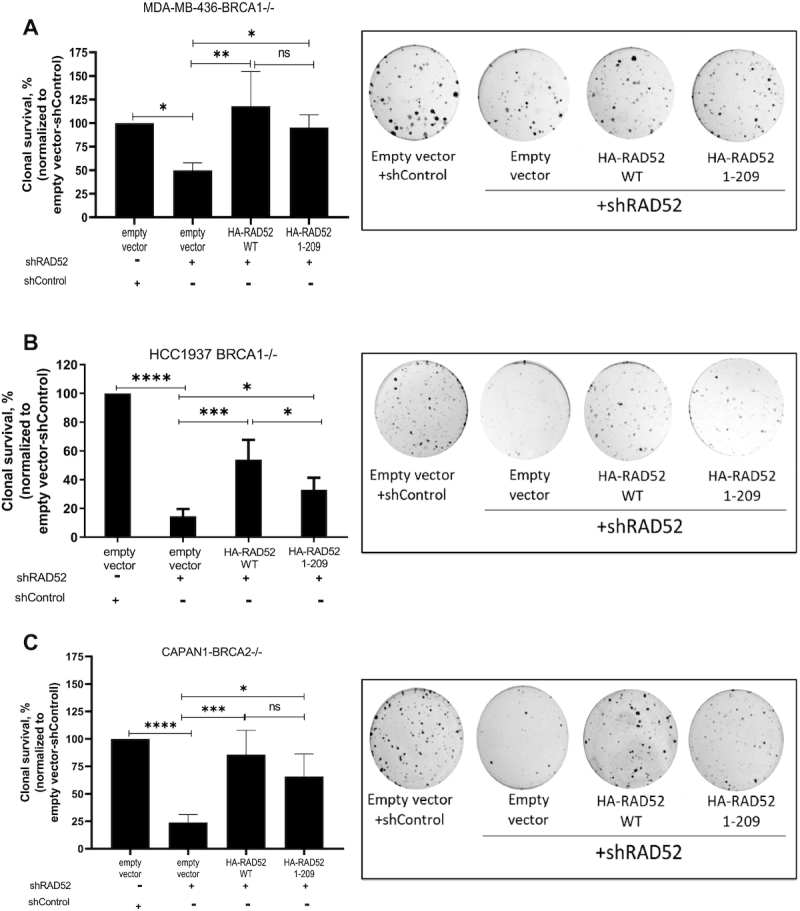
Effect of RAD52_WT_ or RAD52_1–209_ expression on viability of BRCA1- and BRCA2-deficient cells. BRCA1^−/−^ MDA-MB-436 (**A**), HCC1937 (**B**) and *BRCA2*^−/−^ CAPAN1 (**C**) cells expressing RAD52_WT_ or RAD52_1–209_ or empty vector were treated with shRAD52 targeting 3′UTR. After 14 days, the number of colonies were counted. Clonal survival is expressed in percentage after normalizing to cells expressing the empty vector and control shRNA (shCtrl). Clonogenic survival graph are shown on the left and representative crystal violet stained colonies are shown on the right for each panel. Error bars indicate S.D. (*n* = 4) and statistical analysis was performed using one-way ANOVA with Tukeys multiple comparison test; ns *P* > 0.05, *P ≤ 0.05, **P ≤ 0.01, ***P ≤ 0.001 and ^****^P ≤ 0.0001.

Previously, it was demonstrated that RAD52 is essential for viability of human/mammalian cells carrying mutations in *BRCA1*, *BRCA2* and several other HR genes (22,23,32). Downregulating RAD52 by siRNA or inhibiting it with small molecule compounds strongly diminished growth of BRCA-deficient cells (18,33). Here, we tested whether RAD52_1–209_ is sufficient to maintain the viability of BRCA-deficient cells. RAD52_1–209_ was stably expressed in *BRCA1^−^^/^^−^* triple-negative breast cancer cells, MDA-MB-436 and HCC1937, using lentiviral system. First, using the colony formation assay we showed that targeting 3′UTR of endogenous *RAD52* with shRNA (Supplementary Table S1) reduced the viability of MDA-MB-436 and HCC1937 cells approximately 2- and 5-fold, respectively (Figure 2A and B; Supplementary Figure S2A and B). However, the inhibitory effect of shRNA on clonal survival of these cells was rescued by expressing of either RAD52_WT_ or RAD52_1–209_. In the MDA-MB-436 cells, the rescue by RAD52_1–209_ was nearly as robust as that by RAD52_WT_, whereas in HCC1937 cells the rescue effect by RAD52_1–209_ was significant but partial, ∼1.5-fold lower than by RAD52_WT_ (Figure 2A and B).

Similarly, RAD52_1–209_ expression rescued the viability in *BRCA2^−^^/^^−^* CAPAN-1 pancreatic cancer cells, which was reduced approximately 4-fold by treatment with RAD52 shRNA. In these cells, the rescue effect of RAD52_1–209_ was nearly as strong as that of RAD52_WT_ (Figure 2C; Supplementary Figure S2C). These results indicate that expression of RAD52_1–209_ is sufficient to maintain the viability in both *BRCA1*^−/−^ and *BRCA2^−^*^/−^ cells after downregulation of endogenous RAD52.

### Construction and characterization of RAD52 DNA-binding deficient mutants

**Figure 3.**
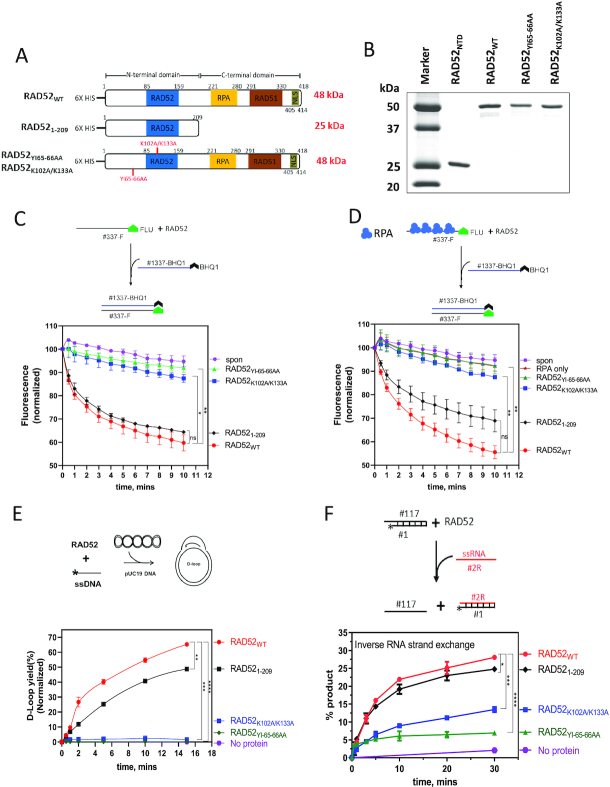
Characterization of the biochemical activities of RAD52_WT_, RAD52_1–209,_ RAD52_YI65–66AA_ and RAD52_K102A/K133A_ proteins. (**A**) The scheme of RAD52 constructs similar to Figure 1A except 6× His tag was added at their N-terminus. RAD52_1–209_ contains 1–209 residues._,_ RAD52_YI65–66AA_ and RAD52_K102A/K133A_ are generated by mutating Y65/I66 and K102/K133 to alanine residues, respectively. (**B**) A 15% SDS-PAGE gel of purified proteins (1 μg each) stained with coomassie blue. Molecular weight markers shown in lane 1. (C and D) Top: the scheme of the ssDNA annealing assay without (**C**) and with RPA (**D**). Fluorescently labeled ssDNA (no. 337-F; 0.5, nM molecules) was pre-incubated with RPA dilution buffer (**C**) or RPA (1 nM) (**D**). RAD52_WT_ (10 nM), RAD52_1–209_ (40 nM), RAD52_YI65–66AA_ (10 nM) or RAD52_K102A/K133A_ (10 nM) was then added to the reaction, followed by addition of complimentary ssDNA carrying a quencher (no. 1337-BHQ; 0.5 nM molecules). Bottom: the kinetics of ssDNA annealing. (**E**) Top: the scheme of the D-loop assay. The asterisk denotes ^32^P-label. RAD52_WT_ (450 nM), RAD52_1–209_ (800 nM), RAD52_YI65–66AA_ (450 nM) or RAD52_K102A/K133A_ (450 nM) was incubated with ssDNA (no. 160; 3 μM, nucleotide) followed by addition of supercoiled pUC19 dsDNA (50 μM, nucleotide) Bottom: the kinetics of D-loop formation. (**F**) Top: the scheme of inverse RNA strand exchange. Asterisk represents ^32^P-label. RAD52_WT_ (900 nM), RAD52_1–209_ (1.4 μM), RAD52_YI65–66AA_ (900 nM), RAD52_K102A/K133A_ (900 nM) was incubated with the 3′-tailed DNA (no. 1/no. 117; 68.6 nM molecules) followed by addition of RNA (no. 2R; 205.8 nM molecules). Bottom: the kinetics of inverse RNA strand exchange. Oligonucleotide sequences for C, D, E and F are shown in Supplementary Table S1. Error bars indicate S.E.M. (*n* = 3) and statistical analysis was performed using Two-way ANOVA with Dunnette's multiple comparison test; ns *P* > 0.05, *P ≤ 0.05, **P ≤ 0.01, ***P ≤ 0.001 and ^****^P ≤ 0.0001. Significance shown for last time-point in C, D, E and F.

The NTD of RAD52 is responsible for ssDNA and RNA annealing and strand exchange, both forward and inverse; it contains the RAD52 primary and secondary DNA binding sites and the region responsible for RAD52 oligomerization, which are required for these reactions (9,12,34,35). Hence, in order to test whether the NTD activities indeed are essential for survival of BRCA-deficient cells, we wanted to generate the RAD52 NTD mutants defective in these activities but retaining an intact CTD and test the effect of these mutants in BRCA-deficient cells. We created two mutants in full-length RAD52 protein (Figure 3A). In the RAD52_YI65–66AA_ mutant, Tyr65 and Ile66 aa residues were substituted with alanines. Previous studies demonstrated that Tyr65 and Ile66 aa residues are critical for ssDNA binding and are located in the primary DNA-binding site (34,36). In the RAD52_K102A/K133A_ mutant, Lys102 and Lys133 aa residues were substituted with alanines. The Lys102 and Lys133 aa residues are important for dsDNA binding and located in the secondary RAD52 DNA binding site (10,34). Next, we purified these mutant proteins (Figure 3B) and characterized them *in vitro* to confirm deficiencies in their biochemical activities.

First, we tested these proteins in the ssDNA annealing activity of the proteins using fluorescently labeled ssDNA (no. 337-F) and its ssDNA complement carrying a Black Hole Quencher group (no.1337-BHQ). Both RAD52_YI65–66AA_ and RAD52_K102A/K133A_ mutants showed nearly no ssDNA annealing activity, whereas RAD52_WT_ and RAD52_1–209_ showed strong activities (Figure 3C)_._ We found that a 2–4-fold higher molar concentration of RAD52 NTD comparing with the RAD52_WT_ full length was required for the optimal ssDNA annealing activity. We suggest that higher RAD52_1–209_ concentration was needed to compensate for its inability to form higher order structures composed of multiple rings which may play a role in RAD52 DNA/RNA pairing (37). We also tested ssDNA annealing activity of these proteins in the presence of RPA that was pre-incubated with ssDNA (no. 337-F) before adding RAD52 proteins (Figure 3D). RPA did not show any effect on the annealing activities of RAD52_WT_ or the RAD52_YI65–66AA_ and RAD52_K102A/K133A_ mutants but marginally inhibited RAD52_1–209_ (Figure 3C and D). Next, we measured DNA strand exchange activity using the D-loop assay. Using ^32^P-labeled ssDNA (no.160) and homologous supercoiled pUC19 plasmid dsDNA, we found that both RAD52_YI65–66AA_ and RAD52_K102A/K133A_ were completely inactive in this assay (Figure 3E and Supplementary Figure S3A). In contrast, both RAD52_WT_ and RAD52_1–209_ were active in the D-loop assay, with a slightly lower activity of RAD52_1–209_. Similarly, we tested RAD52 mutant proteins for inverse RNA strand exchange (IRSE) activity. The nucleoprotein complexes were assembled by incubating RAD52 mutants with 3′-tailed dsDNA (nos.117/1) and IRSE was initiated by adding homologous RNA (no. 2R). As compared to RAD52_WT_ and RAD52_1–209_, RAD52_K102A/K133A_ was partially defective, whereas RAD52_YI65–66AA_ was almost completely defective in IRSE (Figure 3F and Supplementary Figure S3B). Thus, our results show that RAD52_1–209_ possesses similar biochemical activities as RAD52 full-length protein, whereas the RAD52 mutants RAD52_YI65–66AA_ and RAD52_K102A/K133A_ were partially or completely defective in these activities.

### RAD52_1-209_ can promote single-strand DNA annealing (SSA) in human cells

**Figure 4.**
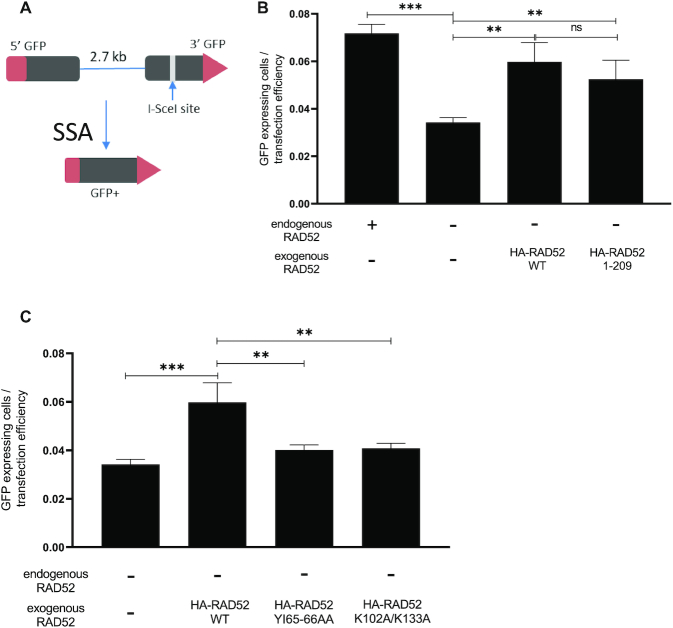
RAD52_1–209_ promotes ssDNA annealing (SSA) in U2OS cells. (**A**) The scheme of the SSA-GFP reporter system. (**B** and **C**) Efficiency of SSA in U2OS cells with endogenous RAD52 knocked out and indicated exogenous RAD52 constructs were expressed. Error bars indicate S.D. (*n* = 3) and statistical analysis was performed using one-way ANOVA with Tukeys multiple comparison test; ns *P* > 0.05, **P ≤ 0.01 and ***P ≤ 0.001.

Previously, it was shown that RAD52 promotes ssDNA annealing (SSA) both in human and yeast cells (6,38). Since the RAD52 NTD retains ssDNA annealing *in vitro*, we suggested that it can also replace WT RAD52 protein in SSA DNA repair pathway in human cells. To measure SSA, we used U2OS cells with the chromosomally integrated SSA-GFP construct. The SSA-GFP reporter system contains a 5′ fragment of the GFP gene, and a 3′ fragment of the GFP (3′-GFP) separated by 2.7 kb region. The 3′ fragment contains an I-SceI endonuclease site. Transfection of cells with the I-SceI expressing vector induces a double-strand break in the 3′-GFP, and its repair by SSA pathway leads to restoration of the GFP gene and its expression detectable by flow cytometry (Figure 4A).

To test the activity of the RAD52 mutants in the SSA pathway, we knocked out endogenous RAD52 by using CRISPR-cas9 in U2OS-SSA cells and then stably integrated into these cells exogenous HA-tagged RAD52_1–209_, RAD52_YI65–66AA_, RAD52_K102A/K133A_ or RAD52_WT_ as a control (Supplementary Figures S1B and S4A). In cells where the endogenous RAD52 was knocked out, the frequency of GFP^+^ cells was reduced ∼2-fold, consistent with the role of RAD52 in DSB repair by SSA (Figure 4B). As expected, expression of exogenous RAD52_WT_ in RAD52 KO cells, rescued the ability of the cells to repair DSBs by SSA (Figure 4B). Importantly, the expression of RAD52_1–209_ also caused ∼1.5-fold increase in SSA in cells with endogenous RAD52 KO (Figure 4B), but both RAD52_YI65–66AA_ and RAD52_K102A/K133A_ were inefficient in rescuing the RAD52 KO effect as compared to RAD52_WT_. Both RAD52_YI65–66AA_ and RAD52_K102A/K133A_ showed no significant increase in SSA compared to RAD52 KO cells (Figure 4C). These data show that intact N-terminal domain of RAD52 (RAD52_1–209_) retains the SSA activity in human cells whereas the mutants of the RAD52 full length protein, carrying mutations in the RAD52 NTD, (RAD52_YI65–66AA_) or (RAD52_K102A/K133A_), lost the SSA activity.

### RAD52_1-209_ can promote Homology-Directed DSB repair (HDR) in *BRCA1*^−/−^ cells

**Figure 5.**
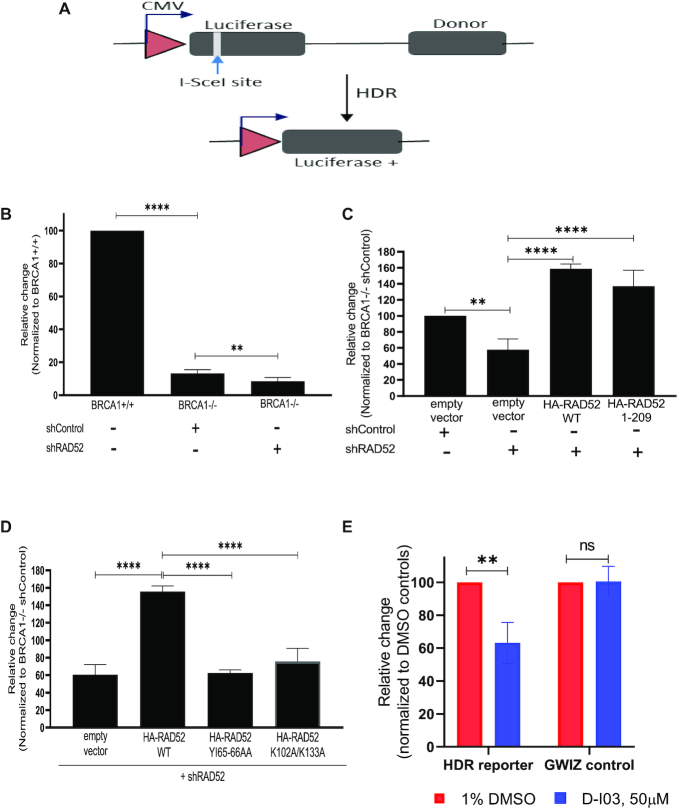
RAD52_1–209_ promotes homology dependent repair (HDR) of DSBs by gene conversion in BRCA1-deficient MDA-MB-436 cells. (**A**) The scheme of the HDR assay. (**B**) Efficiency of I-Sce induced HDR in MDA-MB-436 *BRCA1*^+/+^ and MDA-MB-436 *BRCA1*^−/−^ treated with shRAD52 or a control shRNA (shCtrl). The relative change of HDR efficiency for each group was calculated by normalizing each to the HDR efficiency obtained for BRCA1^+/+^ cells. (**C**) Efficiency of I-Sce induced HDR in BRCA1^−/−^ cells expressing RAD52_WT_ or RAD52_1–209_ or empty vector treated with shRAD52 or control shRNA(shCtrl). (**D**) Efficiency of I-Sce induced HDR in *BRCA1*^−/−^ cells expressing RAD52_WT_ or RAD52_YI65–66AA_ or RAD52_K102A/K133A_ or empty vector treated with shRAD52.The relative change of HDR efficiency for each group in panels (C) and (D) was calculated by normalizing to the HDR efficiency obtained for cells expressing empty vector and shCtrl. (**E**) Efficiency of I-Sce induced HDR in *BRCA1*^−/−^ cells expressing RAD52_1–209_ with shRAD52 and treated with D-I03 (50 μM) or DMSO (1%). Data normalized to the HDR efficiency obtained for cells treated with DMSO. Error bars represent S.D (*n* = 3), statistical analysis was performed using One-way ANOVA with Tukeys multiple comparison test in (B), (C) and (D) and Two-way ANOVA with Bonferroni's multiple comparisons test in (E); **P ≤ 0.01, ^****^P ≤ 0.0001 and ns *P* > 0.05.

Previously, it was proposed that RAD52 supports viability of BRCA-deficient cells by stimulating the RAD51-dependent HDR (22,23). Furthermore, it was suggested that in BRCA-deficient cells this stimulation may involve the mediator function of RAD52, i.e. loading of RAD51 on RPA-covered ssDNA. Alternatively, one can suggest that RAD52 promotes HDR through its DNA strand exchange (D-loop formation) activity (Figure 3C). Here, we tested whether RAD52_1–209_ that is devoid of RAD51- and RPA-interaction regions can still mediate HDR in BRCA-deficient cells. To test the HDR activity of RAD52_1–209_ in BRCA1-deficient MDA-MB-436 cells, we used the HDR luciferase construct. This construct consists of two inactive copies of the luciferase gene, one is inactivated by incorporation of I-SceI endonuclease site and another is non-transcribable due to the lack of promoter. When the HDR construct is co-transfected with I-SceI endonuclease expressing plasmid, a unique DSB is generated by I-SceI, which undergoes repair by gene conversion leading to expression of luciferase gene that can be measured using luminometer (Figure 5A). Using this assay, we first tested the HDR in BRCA1-deficient MDA-MB436 cells and in their isogenic BRCA1-reconstituted counterparts. As expected, we noticed a ∼7-fold lower HDR efficiency in *BRCA1*^−/−^ cells compared to BRCA1-reconstituted cells (Figure 5B). When we treated the *BRCA1*^−/−^ cells with anti-RAD52 shRNA, in accord with a previous report we saw a further decrease in HDR efficiency (22,23) (Figure 5B and C). As expected, expression of exogenous RAD52_WT_ in these cells rescued the HDR (Figure 5C). Next, we found that RAD52_1–209_ was also able to rescue the effect of the RAD52 knock-down, nearly with the same efficiency as the RAD52_WT_ (Figure 5C). In contrast, RAD52_YI65–66AA_ or RAD52_K102A/K133A_ mutants did not rescue HDR in MDA-MB-436 BRCA-deficient cells in which endogenous RAD52 was knocked down with shRNA (Figure 5D).

We also tested whether the HDR rescued in BRCA1-deficient MDA-MB-436 cells by RAD52_1–209_ expression was sensitive to RAD52 inhibitor D-I03 (8,39). First, in a separate experiment, we showed that *in vitro* D-I03 targets the D-loop formation activity of RAD52_1–209_ with same efficiency as that of RAD52_WT_ (Supplementary Figure S5). Then we found that treatment with D-I03, reduced the number of luciferase^+^ cells only in cells transfected with HDR construct and not in cells transfected with a control luciferase encoding plasmid gWIZ (Figure 5E).

Thus, our current results show that the DNA pairing activity of RAD52_1–209_ is sufficient for the rescue of HDR in BRCA^−/−^ cells. These results suggest that in BRCA^−/−^ cells the RAD52 role in HDR is independent of its potential RAD51-mediator function.

### RAD52_1-209_: DNA-binding domain is essential for its role in cells

**Figure 6.**
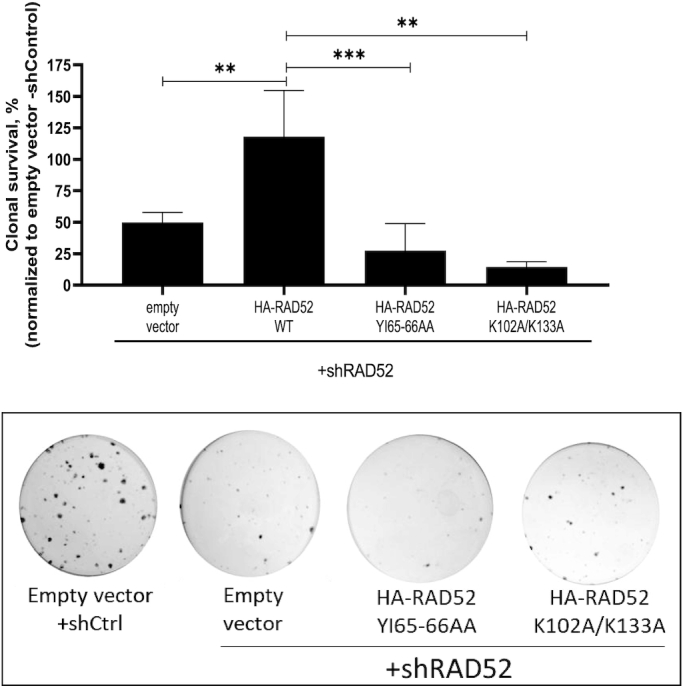
The RAD52_YI65–66AA_ and RAD52_K102A/K133A_ mutants are incapable to maintain survival of *BRCA1*^−/−^ MDA-MB-436 cells. Top: Clonogenic survival graph; bottom: representative crystal violet stained colonies are shown on the right for each panel. Error bars represent mean ± S.D. (*n* = 3) and statistical analysis was performed using one-way ANOVA with Tukeys multiple comparison test; **P ≤ 0.01 and ***P ≤ 0.001.

**Figure 7.**
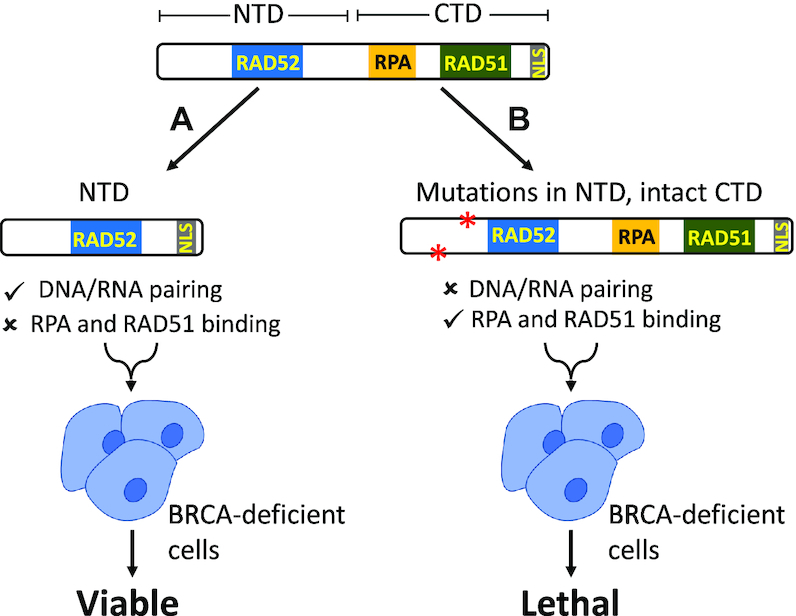
The function of RAD52 N-terminal domain in BRCA-deficient cells. (**A**) The DNA/RNA pairing activities of RAD52 possessed by NTD is essential for viability of BRCA-deficient cells. (**B**) NTD mutants deficient in DNA/RNA pairing activities cause cell death.

Next, we wanted to test the effect of RAD52_YI65–66AA_ and RAD52_K102A/K133A_ mutants on viability of *BRCA*^−/−^ MDA-MB-436 cells. Using colony formation assay we found that both these mutants could not rescue viability of cells in which endogenous RAD52 was inhibited by shRNA. Cells expressing RAD52_YI65–66AA_ and RAD52_K102A/K133A_ showed lower clonal survival, approximately 5- and 7-fold, respectively, than cells expressing RAD52_WT_. When compared with empty vector transfectants, these mutants showed no significant effect on clonal survival (Figure 6A and Supplementary Figure S4A). We also, tested the ability of these two mutants to form foci, upon cisplatin treatment. Both RAD52_YI65–66AA_ and RAD52_K102A/K133A_ were defective in forming foci as compared to RAD52_WT_ or RAD52_1–209_ (Supplementary Figure S4B and S4C). Taken together, our data demonstrates that the DNA pairing activities residing in the RAD52 NTD are essential for the viability of BRCA-deficient cells (Figure 7).

## DISCUSSION

Discovery of the synthetic lethality relationship between RAD52 and BRCA raised a major question on the role of RAD52 in both normal and BRCA-deficient cells (25,33). Yeast Rad52 that plays a major role in DNA repair and HR possesses two major activities: (i) it has ssDNA and RNA annealing and RNA/DNA pairing activities and (ii) it acts as a mediator by facilitating loading of Rad51 on ssDNA in the presence of RPA. Human RAD52 retains DNA/RNA annealing and pairing activities but was presumed to play only a minor role in HR because its mediator function was conceded to BRCA2. However, while the mediator activity of human RAD52 was not demonstrated so far in the biochemical studies, it cannot be ruled out. Similar to yeast orthologue, human RAD52 can physically interact with human RAD51 and RPA, a minimal attribute of the mediator function that may escape detection *in vitro* due to missing post-translational RAD52 modifications or auxiliary proteins (40).

Previously, it was shown that the NTD (1–209 residues) of RAD52 which comprises the DNA binding region and protein oligomerization domain is capable of performing all DNA/RNA pairing activities of RAD52 *in vitro* (18,24). On the other hand, it was shown that the RAD52 CTD is responsible for binding to RPA and RAD51 (41,42) taking advantage of this separation of functions between the RAD52 domains, we aimed to understand which of these two major RAD52 activities, DNA/RNA pairing or mediation of RAD51 ssDNA binding, is essential for viability of BRCA-deficient human cells.

In order to address this question, we inactivated endogenous RAD52 in BRCA-deficient cells by shRNA and replaced it with RAD52_1–209_. RAD52 possesses a nuclear localization signal (NLS) located at the C-terminus, which is essential for transporting of RAD52 from the cytoplasm to the nucleus (43). Deletion of the NLS results in retention of RAD52 in the cytoplasm and in reduction of SSA, the hallmark RAD52 activity in the cell (44). Therefore, to maintain the cellular functionality of RAD52_1–209_, we fused this construct with residues (405–418) from CTD comprising the NLS (405–414) (Figure 1A). We found that this RAD52_1–209_ construct was functional in human cells; it formed nuclear foci in response to DNA damage and was able to promote DSB repair by the HDR and SSA pathways (Figure 1B, Supplementary Figure S1B, 4B and 5C).

Importantly, our results demonstrated that the RAD52_1–209_ is sufficient to maintain cell viability of BRCA1- and BRCA2-deficient cells (Figure 2). In contrast, the RAD52_Y65–66AA_ and RAD52_K102A/K133A_ mutants of the RAD52 full-length protein, deficient in these activities either fully or partially, were unable to rescue the viability of BRCA-deficient cells in which endogenous RAD52 was depleted (Figure 6). This result accentuates the essential role of the RAD52 NTD in viability of BRCA1&2-deficient cells.

The RAD52 NTD encodes several activities including ssDNA and RNA annealing, D-loop formation, and inverse RNA strand exchange. We wanted to examine the effect of DNA binding deficient mutants RAD52_Y65–66AA_ and RAD52_K102A/K133A_ on the RAD52 activities and cellular functions. Both RAD52_Y65–66AA_ and RAD52_K102A/K133A_ completely lost the DNA pairing (D-loop formation) activity *in vitro*, which was consistent with their deficiency in DSB repair through the HDR mechanism in BRCA-deficient human cells (Figures 3E and 5D). Both mutants also showed deficiency in ssDNA annealing activity *in vitro*, consistent with previous studies (10) (Figure 3C and D). As RAD52 ssDNA annealing activity is required for DSB repair by the SSA mechanism, these mutants were deficient in SSA in human cells (Figure 4C). Similarly, both RAD52_Y65–66AA_ and RAD52_K102A/K133A_ were deficient in inverse RNA strand exchange, with a stronger deficiency observed for RAD52_Y65–66AA_ (Figure 3F). Since both these mutants are unable to rescue viability of BRCA-deficient cells upon endogenous RAD52 depletion, these results support our conclusion that the DNA/RNA pairing and/or annealing activities of RAD52/RAD52 NTD are important for viability of BRCA-deficient cells. Additional studies are needed to determine which of these activities plays the most important role in BRCA-deficient cells.

In previous studies, it was shown that RAD52 is responsible for the residual HDR in BRCA-deficient human cells. It was suggested that the HDR activity of RAD52 may be attributed to its potential mediator function (22,23,39). Our results show that RAD52_1–209_, devoid of the RAD51 and RPA binding regions and therefore incapable of performing the mediator function, can still carry out HDR in BRCA-deficient cells (Figure 5C). These results suggest that the DNA pairing (D-loop formation) activity of RAD52 is responsible for HDR in BRCA-deficient cells rather than its mediator function. Our results with RAD52 small molecule inhibitor D-I03 corroborate this conclusion. D-I03 inhibits RAD52_1–209_ D-loop formation activity *in vitro* and also HDR promoted by the RAD52 NTD in BRCA-deficient cells (Figure 5C and Supplementary Figure S5).

RAD52 CTD possesses the RPA- and RAD51-interacting regions, and our results with RAD52_1–209_ may raise a query regarding the role of these interactions for the RAD52 cellular function. Apart from above mentioned functions, recent reports show that RAD52 plays a role in various pathways of DNA repair including break-induced replication, transcription-coupled HR, replication fork restart, and alternative lengthening of telomeres (25,45–49). One may suggest that RAD52 interaction with RPA and RAD51 may be important for these functions of RAD52, but further studies are needed for better understanding of the role of these interactions.

Taken together, we have shown that the NTD of RAD52 plays a pivotal role in the RAD52 cellular functions and in maintaining viability of BRCA-deficient cells (Figure 7). These findings designate the RAD52 NTD as a potential therapeutic target in BRCA-deficient tumors.

## DATA AVAILABILITY

The data that support the findings of this study are presented in the main text paper and in the online Supplementary Information file.

## Supplementary Material

gkaa1145_Supplemental_File
