# Supplementary material for: The function of RAD52 N-terminal domain is essential for viability of BRCA-deficient cells
Source: Nucleic Acids Res. 2020 Dec 4;48(22):12778–91. doi: 10.1093/nar/gkaa1145 (PMC7736796; doi:10.1093/nar/gkaa1145)
Supplement: gkaa1145_Supplemental_File [file gkaa1145_supplemental_file.pdf]

Supplementary information

The essential function of RAD52 protein for viability of BRCA-deficient cells

Kritika Hanamshet and Alexander V. Mazin.

Department of Biochemistry and Molecular Biology, Drexel University College of Medicine, Philadelphia, PA 19102, USA.

*Corresponding Author:
Alexander Mazin, Ph. D.
Drexel University College of Medicine
Department of Biochemistry and Molecular Biology
245 N 15th Street, MS 497, NCB, Room 10103
Philadelphia, PA 19102–1192
Tel: 215–762–7195; Fax: 215–762–4452
E-mail: avm28@drexel.edu

Contents:

1. Supplementary Figures S1-S5

2. Supplementary Table S1

Figure S1

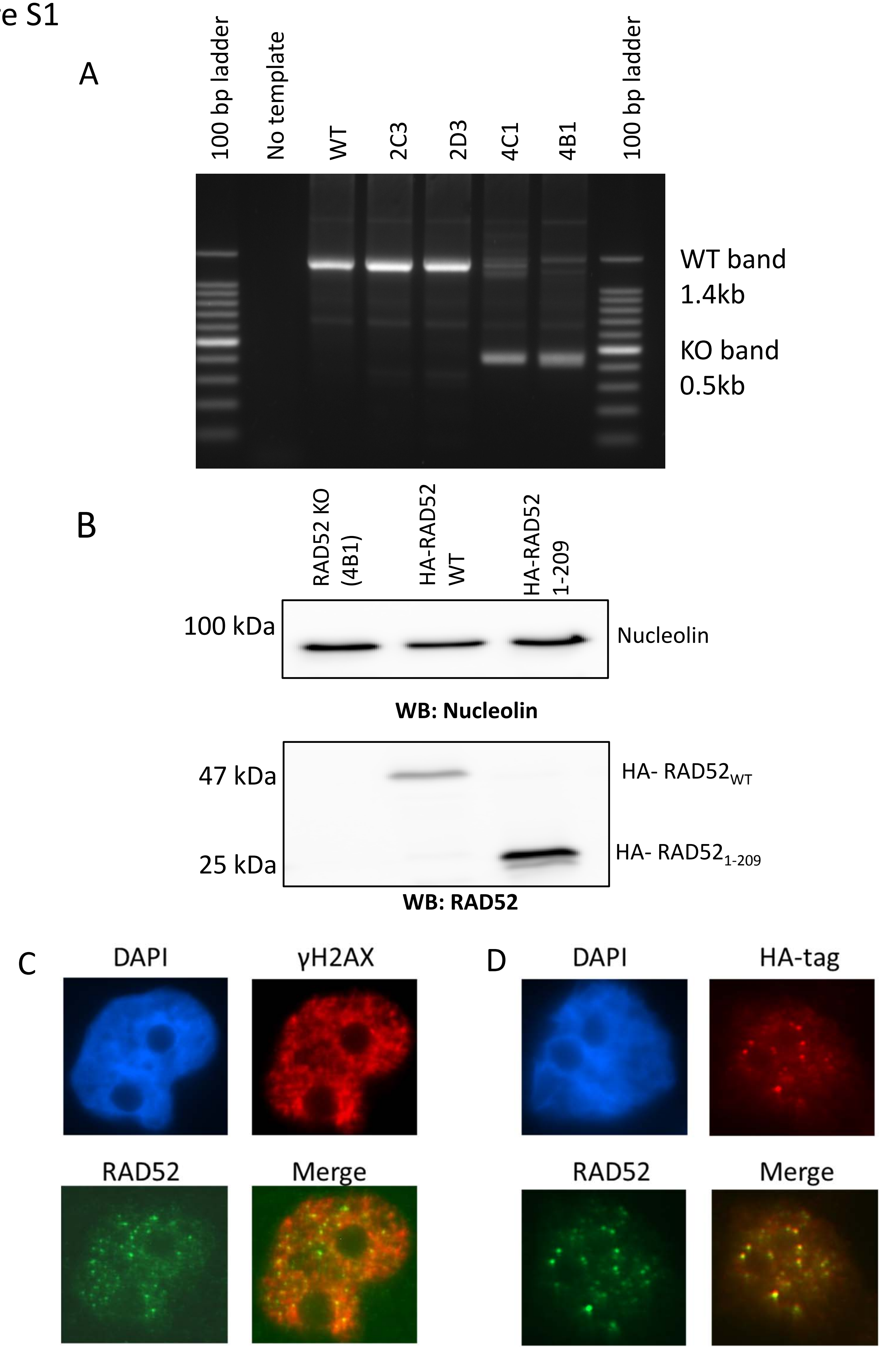

MDA-MB-436 cells expressing shRAD52 and exogenous HA-RAD52$_{1-209}$

Figure S1

RAD52$_{1-209}$ forms foci in response cisplatin treatment. (A) Knock-out of *RAD52* by CRISPR-Cas9 in different U2OS cell clones was confirmed by PCR. (B) The expression of exogenous HA-RAD52$_{WT}$ or HA-RAD52$_{1-209}$ proteins in 4B1 U2OS cell clone was confirmed by Western blot. (C) and (D) Representative images of MDA-MB-436 cells stably expressing RAD52$_{1-209}$ and shRAD52, stained 6 h after cisplatin (10 μM) treatment with DAPI (blue), or probed with fluorescent anti-bodies for RAD52 (green), γ-H2AX (red) in (C), and RAD52 (green), HA-tag (red) in (D).

Figure S2

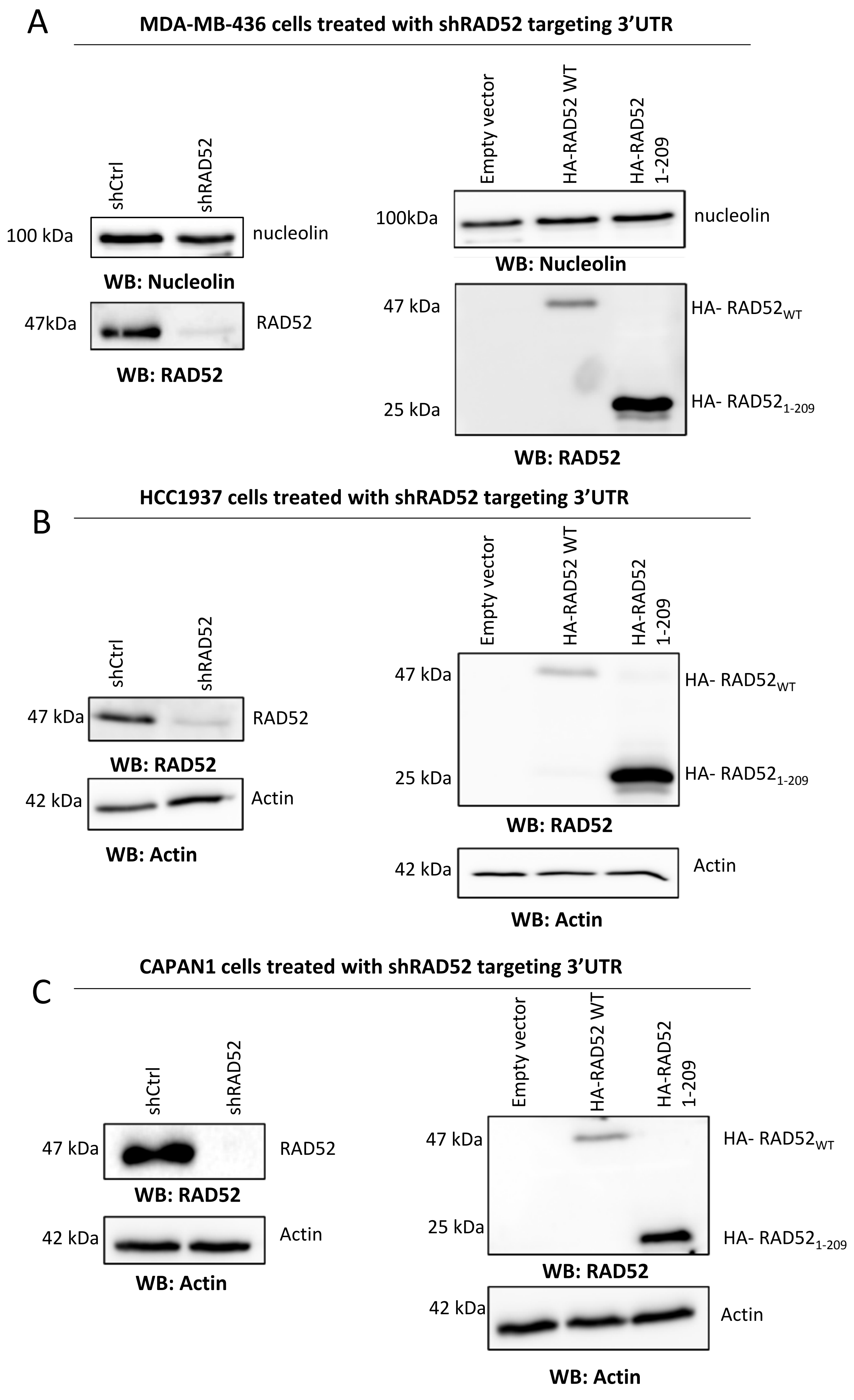
A
MDA-MB-436 cells treated with shRAD52 targeting 3'UTR
shCtrl
shRAD52
100 kDa
nucleolin
WB: Nucleolin
47kDa
RAD52
WB: RAD52
Empty vector
HA-RAD52 WT
HA-RAD52 1-209
100kDa
nucleolin
WB: Nucleolin
47 kDa
HA- RAD52WT
25 kDa
HA- RAD521-209
WB: RAD52
B
HCC1937 cells treated with shRAD52 targeting 3'UTR
shCtrl
shRAD52
47 kDa
RAD52
WB: RAD52
42 kDa
Actin
WB: Actin
Empty vector
HA-RAD52 WT
HA-RAD52 1-209
47 kDa
HA- RAD52WT
25 kDa
HA- RAD521-209
WB: RAD52
42 kDa
Actin
WB: Actin
C
CAPAN1 cells treated with shRAD52 targeting 3'UTR
shCtrl
shRAD52
47 kDa
RAD52
WB: RAD52
42 kDa
Actin
WB: Actin
Empty vector
HA-RAD52 WT
HA-RAD52 1-209
47 kDa
HA- RAD52WT
25 kDa
HA- RAD521-209
WB: RAD52
42 kDa
Actin
WB: Actin

Figure S2

Western blot analysis of knock-down of endogenous RAD52. Cells treated with shRAD52 and control shRNA (shCtrl) (left panels) and expression of exogenous HA-RAD52$_{WT}$ or HA-RAD52$_{1-209}$ in the cells treated with shRAD52 (right panels) in MD-MB-436 (A), HCC1937 (B), and CAPAN1 (C) cells.

Figure S3

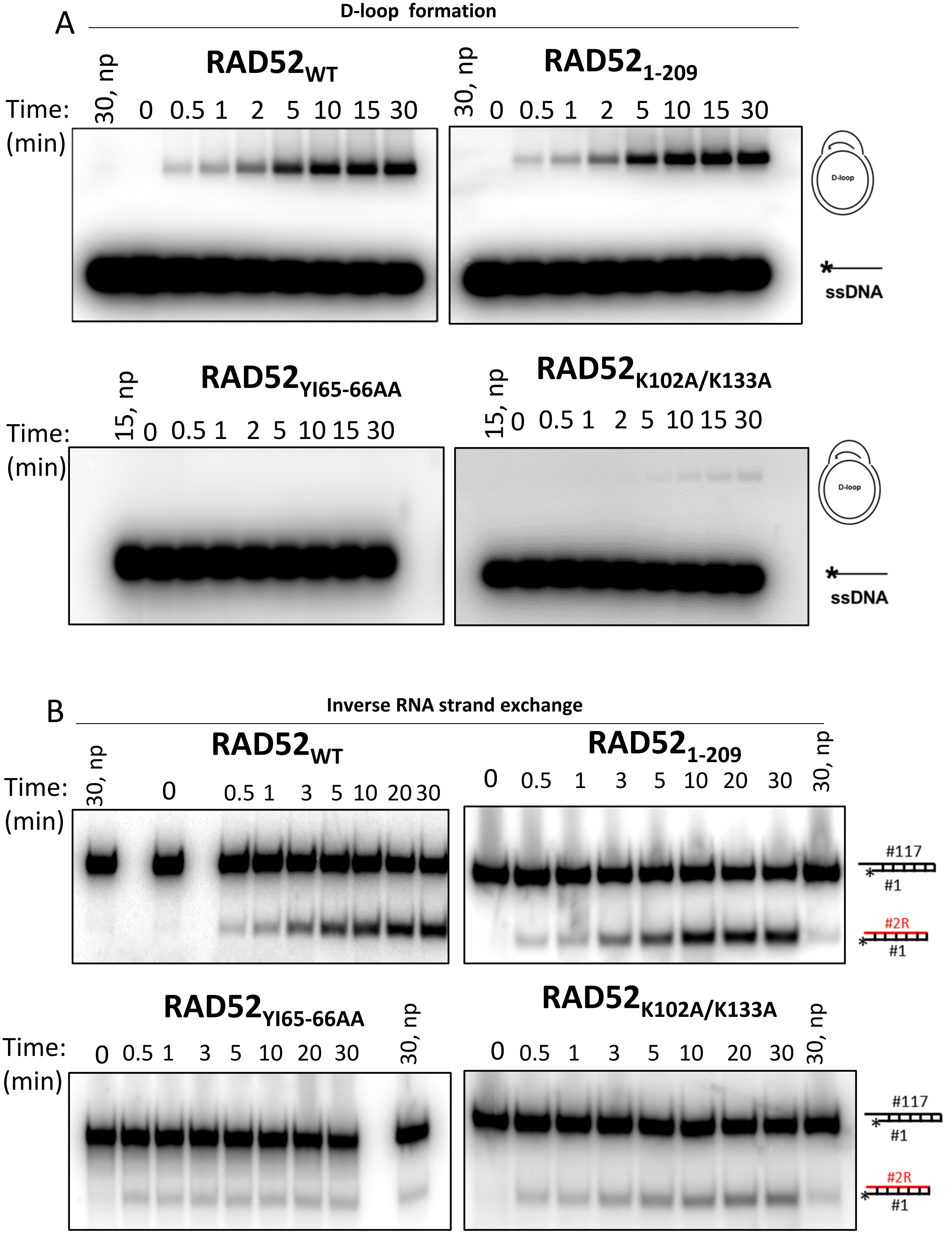

np: no protein

Figure S3

D-loop formation and inverse RNA strand exchange activity of $RAD52_{WT}$ and RAD52 mutants. (A) Representative images of agarose gels containing D-loops formed by for $RAD52_{WT}$, $RAD52_{1-209,}$ $RAD52_{YI65-66AA}$ or $RAD52_{K102A/K133A.}$ The gels were quantified, and the data presented graphically in Figure 3C. (B) Representative images of polyacrylamide gels after inverse RNA strand exchange assay for $RAD52_{WT}$, $RAD52_{1-209,}$ $RAD52_{YI65-66AA}$ and $RAD52_{K102A/K133A.}$ The gels were quantified, and the data presented graphically in Figure 3E. In “np: no protein” control, RAD52 protein was substituted by storage buffer.

Figure S4

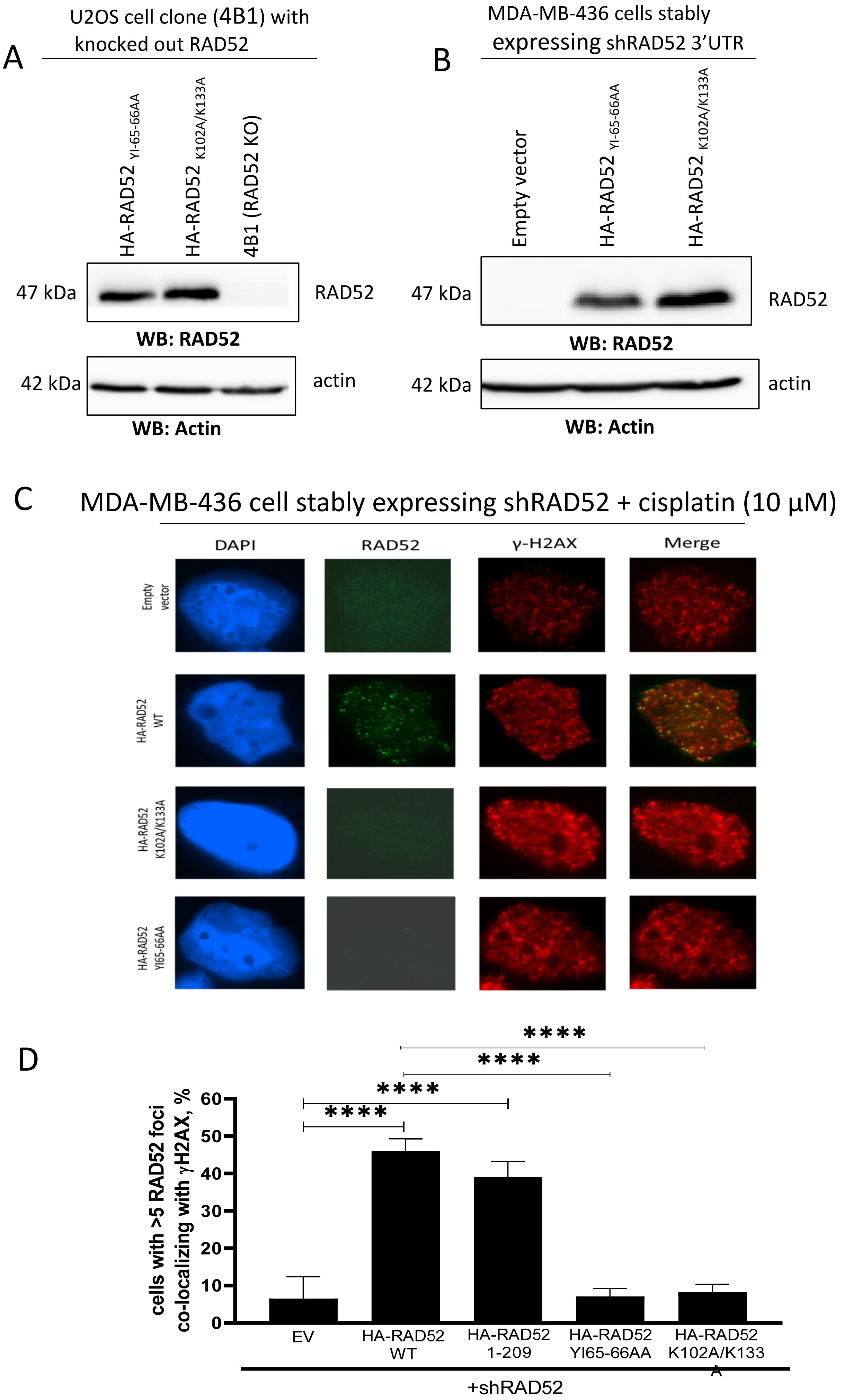

A
U2OS cell clone (4B1) with knocked out RAD52
HA-RAD52 YI-65-66AA
HA-RAD52 K102A/K133A
4B1 (RAD52 KO)
47 kDa
RAD52
WB: RAD52
42 kDa
actin
WB: Actin
B
MDA-MB-436 cells stably expressing shRAD52 3'UTR
Empty vector
HA-RAD52 YI-65-66AA
HA-RAD52 K102A/K133A
47 kDa
RAD52
WB: RAD52
42 kDa
actin
WB: Actin
C
MDA-MB-436 cell stably expressing shRAD52 + cisplatin (10 μM)
DAPI
RAD52
γ-H2AX
Merge
Empty vector
HA-RAD52 WT
HA-RAD52 K102A/K133A
HA-RAD52 YI65-66AA
D
cells with >5 RAD52 foci co-localizing with γH2AX, %
60
50
40
30
20
10
0
****
****
****
****
EV
HA-RAD52 WT
HA-RAD52 1-209
HA-RAD52 YI65-66AA
HA-RAD52 K102A/K133 A
+shRAD52

Figure S4

RAD52$_{YI65-66AA}$ and RAD52$_{K102A/K133A}$ are incapable to form foci in response cisplatin treatment. (A) Western blot confirmation of knocked out of endogenous RAD52 (KO) and expression of exogenous RAD52$_{YI65-66AA}$ and RAD52$_{K102A/K133A}$ in U2OS cells. (B) Western blot confirmation of knocked-down endogenous RAD52 by stable expression of shRAD52 and expression of exogenous HA-RAD52$_{WT}$ or HA-RAD52$_{1-209}$ in MD-MB-436 cells. (C) Representative images of MDA-MB-436 cells stably expressing shRAD52 and RAD52$_{YI65-66AA}$ or RAD52$_{K102A/K133A}$, stained 6 h after cisplatin (10 μM) treatment with DAPI (blue), fluorescent anti-bodies for RAD52 (green) and γ-H2AX (red). (D) Quantification of RAD52 foci co-localized with γ-H2AX foci in MDA-MB-436 cells treated with shRAD52 and stably expressing indicated exogenous RAD52 protein.

Figure S5

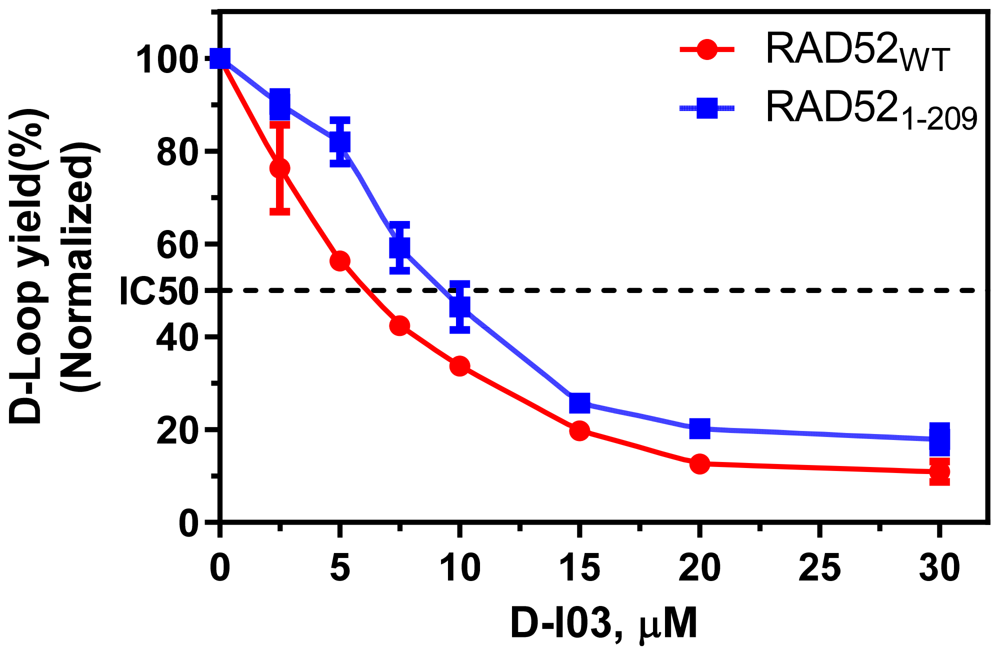

RAD52WT
RAD521-209
100
80
60
IC50
40
20
0
D-Loop yield(%)
(Normalized)
0
5
10
15
20
25
30
D-I03, μM

Figure S5

D-I03 inhibits the D-loop activity of $RAD52_{WT}$ and $RAD52_{1\text{-}209}$ with a similar efficiency.

Table S1: Sequences of the oligonucleotides used in this study.

Oligo name	length	Sequence (5'-3')
160	80	AGCTTTGAGATGCTTGCTTATCAACAGAAGGAGTTACCTCGCGTTGCGTCTATTATGGTA CCACACCAATCTTTCCAAGCAACG
1337-BHQ	39	AGCACTGACTACTGTCGTCGATCATCGTGCATCACAGTG–BHQ1
337-F	60	FLU-CACTGTGATGCACGATGATCGACGACA GTAGTCAGTGCTGGGTCAACATCTGTATGCAGG
1	63	ACAGCACCAGATTCAGCAATTAAGCTCTAAGCCATCCGCAAAAATGACCTCT TATCAAAAGGA
117	94	TCCTTTTGATAAGAGGTCATTTTTGCGGATGGCTTAGAGCTTAATTGCTGA ATCTGGTGCTGTAGGTCAACATGTTGTAAATATGCAGCTAAAG
2R	63	UCCUUUUGAUAAGAGGUCAUUUUUGCGGAUGGCUUAGAGCUUAAUUGCUGAA UCUGGUGCUGU (ribonucleotide)
RAD52 sgRNA L1 up F	25	CACCGCTAGGCTGGAGTCCGACCAG
RAD52 sgRNA L1 up R	25	AAACCTGGTCGGACTCCAGCCTAGC
RAD52 sgRNA R1 down F	25	CACCGACCCACAGCAGACTTTCAGC
RAD52 sgRNA R1 down R	25	AAACGCTGAAAGTCTGCTGTGGGTC
RAD52 check primer Forward	20	AATTCATGTGCCTGGAAAGC
RAD52 check primer Reverse	20	CCCACGTAGAACTTGCCATT
shRAD52 3'UTR Sigma SHCLND-NM_134424.2-1462s21c1	57	CCGGATGAACGTCATTGCGATTTATCTCGAGATAAATCGCAATGACGTTCATTTTTTG
shControl Sigma SHC202	57	CCGGCAACAAGATGAAGAGCACCAACTCGAGTTGGTGCTCTTCATCTTGTTGTTTTT
